# Processing ambiguity in a linguistic context: decision-making difficulties in non-aphasic patients with behavioral variant frontotemporal degeneration

**DOI:** 10.3389/fnhum.2015.00583

**Published:** 2015-10-27

**Authors:** Nicola Spotorno, Meghan Healey, Corey T. McMillan, Katya Rascovsky, David J. Irwin, Robin Clark, Murray Grossman

**Affiliations:** ^1^Penn Frontotemporal Degeneration Center, University of Pennsylvania, Perelman School of MedicinePhiladelphia, PA, USA; ^2^Department of Linguistics, University of PennsylvaniaPhiladelphia, PA, USA

**Keywords:** anaphoric pronouns, decision-making, prefrontal cortex, parietal cortex

## Abstract

Some extent of ambiguity is ubiquitous in everyday conversations. For example, words have multiple meaning and very common pronouns, like “he” and “she” (anaphoric pronouns), have little meaning on their own and refer to a noun that has been previously introduced in the discourse. Ambiguity triggers a decision process that is not a subroutine of language processing but rather a more general domain resource. Therefore non-aphasic patients with limited decision-making capability can encounter severe limitation in language processing due to extra linguistic limitations. In the present study, we test patients with behavioral variant frontotemporal degeneration (bvFTD), focusing on anaphora as a paradigmatic example of ambiguity resolution in the linguistic domain. bvFTD is characterized by gray matter (GM) atrophy in prefrontal cortex, but relative sparing of peri-Sylvian cortex. A group of patients with parietal disease due to corticobasal syndrome (CBS) was also tested here in order to investigate the specific role of prefrontal cortex in the task employed in the current study. Participants were presented with a pair of sentences in which the first sentence contained two nouns while the second contained a pronoun. In the experimental (ambiguous) condition, both nouns are plausible referents of the pronoun, thus requiring decision-making resources. The results revealed that bvFTD patients are significantly less accurate than healthy seniors in identifying the correct referent of a pronoun in the ambiguous condition, although CBS patients were as accurate as healthy seniors. Imaging analyses related bvFTD patients’ performance to GM atrophy in ventromedial prefrontal cortex (vmPFC). These results suggest that bvFTD patients have difficulties in decision processes that involve the resolution of an ambiguity.

## Introduction

Ambiguity can be defined as a property of any concept, sentence or choice that cannot be characterized or solved following a precise rule. Ambiguity is nested in verbal communications as well as in other sorts of everyday interaction. Words like “bank” have multiple meanings and only the context can lead to the most appropriate interpretation. Other words like the anaphoric pronouns have minimal meaning and just refer to a concept that was previously introduced in the discourse. Those pronouns (e.g., “he” or “she”) are extremely common in daily speech and the degree with which the identification of the referent of a pronoun is simple or rather requires a more sophisticate decision process can be easy manipulate in an experimental setting. Consider, for example, the sentence pair:

(1) “John kissed Mary. She smiled.”

The pronoun “she” clearly derives its referent from the antecedent noun “Mary,” which was the only female noun presented in the previous utterance. However, daily communicative exchanges are not always perfect, and listeners and readers might face ambiguities, as in (2):

(2) “John kissed the visitor. He smiled.”

Here, there are two possible antecedents (“John” and “the visitor”). Recent evidence from our group (McMillan et al., 2012a) suggested that a prefrontal network, including dorsolateral prefrontal cortex (dlPFC), inferior frontal gyrus (IFG) and ventromedial prefrontal cortex (vmPFC) supplies the cognitive resources necessary for the decision about the intended referent of the pronoun. Those regions are know in the decision-making literature as hubs of a more general domain network that supports choices in different domains like finance and ethics (see e.g., Casey et al., 2001; Sanfey et al., 2003; Swick et al., 2008; Scheibe et al., 2010; Rilling and Sanfey, 2011).

In the present study, we aim to employ instances of ambiguous anaphoric pronouns as a key study for investigating decision-making limitations in a group of non-aphasic patients with behavioral variant frontotemporal degeneration (bvFTD).

bvFTD is a neurodegenerative disease associated with progressive frontal and anterior—inferior temporal atrophy that results in inappropriate social behavior and executive difficulty (Rascovsky et al., 2011; Mendez et al., 2014). Imaging studies (Gregory et al., 2002; Kipps and Hodges, 2006; Eslinger et al., 2007; Grossman, 2007; Adenzato et al., 2010; Grossman et al., 2010) and autopsy reports (Hu et al., 2007; Brettschneider et al., 2014) have shown that bvFTD affects dorsolateral, ventral and medial regions of the frontal lobe. These prefrontal regions have been implicated in functional MRI (fMRI) studies of social cognition (e.g., Amodio and Frith, 2006; Frith and Frith, 2006; Saxe, 2006; Mitchell, 2009; Van Overwalle and Baetens, 2009; Enrici et al., 2011) and reasoning (e.g., Bhatt and Camerer, 2005; Rypma et al., 2005; Goel, 2007; Prado and Noveck, 2007; Coricelli and Nagel, 2009; Bhatt et al., 2010; Prado et al., 2015). Previous work has also linked deficits in social coordination in bvFTD patients to gray matter (GM) atrophy in dlPFC, rostral prefrontal cortex (rPFC) and vmPFC (McMillan et al., 2012b; Healey et al., 2015).

To evaluate the specificity of the frontal contributions to the decision process, we also evaluated a control group with corticobasal syndrome (CBS), which presents with minimal frontal disease burden. CBS is a neurodegenerative condition characterized by progressive atrophy that is more prominent in the parietal cortices and typically extends into dorsolateral portions of the frontal lobe in more advanced stages. CBS patients have an asymmetric extrapyramidal syndrome involving features such as limb rigidity, dystonia and “alien limb” phenomena, together with progressive ideomotor apraxia and cortical sensory loss (Boeve et al., 1999; Grimes et al., 1999; Murray et al., 2007; Armstrong et al., 2013; Alexander et al., 2014). We identified CBS patients without progressive aphasia and without a disorder of behavioral conduct and in addition to that, we select CBS patients in the mild stage of the disease in order to reduce the probability of including patients with significant prefrontal atrophy (see “Materials and Methods” Section).

We presented both patient groups and an additional group of healthy seniors with a decision task in which the participants are required to identify the correct or most likely referent of an anaphoric pronoun. In cases such as: (1) we expected that the bvFTD patients would be able to correctly identify the antecedent of the pronoun as well as healthy seniors and CBS patients because the linguistic code provides sufficient information. This finding would be consistent with the relatively spared language ability typically seen in bvFTD patients. However, we expected that bvFTD would exhibit significant difficulty in respect to healthy seniors and CBS patients in identifying referent of the pronoun in ambiguous cases, such as; (2) which places additional demands on decision-making abilities. We also predict that difficulties in the resolution of the ambiguous trials will correlate with cortical thinning in prefrontal areas. For example, increasing activation in vmPFC during economic games has been associated with the processing of uncertainty when the probabilities are known (see e.g., Huettel et al., 2005). In our case, both the context and participants’ experience with anaphoric pronouns provide them with information about the likelihood that one noun or the other is most plausible referent. Atrophy in vmPFC may affect bvFTD patients’ ability to evaluate and connect the specific instance of ambiguous choice with their knowledge about uncertainty.

## Materials and Methods

### Participants

We examined 23 patients with neurodegenerative disease, including patients with bvFTD (*N* = 13) and CBS (*N* = 10). Patients were recruited from the University of Pennsylvania Health System Cognitive Neurology clinics. Patients were diagnosed by board-certified neurologists (MG, DJI) using published consensus criteria (Rascovsky et al., 2011; for bvFTD and Armstrong et al., 2013; for CBS). Exclusion criteria included a primary psychiatric disorder, structural brain lesion, and encephalopathy due to a medical condition. All patients underwent a screening procedure to ensure the absence of any condition or medication that could compromise cognitive performance. Patients were also presented with the Boston Naming Test (BNT) in order to ensure the absence of basic linguistic deficits in lexical processing. Twelve demographically-comparable healthy seniors were also recruited from the community. Healthy seniors underwent a screening procedure to ensure the absence of any condition or medication that could compromise cognitive performance and had to score 28 or higher (out of 30) on the Mini-Mental State Exam (MMSE). Clinical, demographic, and neuropsychological information are summarized in Table 1. All patients were in the mild stage of their disease as indicated by their MMSE score (average bvFTD: 25.7; average CBS: 27.2; see Table 1). All subjects participated in an informed consent procedure approved by an Institutional Review Board at the University of Pennsylvania.

**Table 1:** **Mean (±SEM) demographic and neuropsychological data for patient and control groups**.

Demographic/Clinical measure	bvFTD (*N* = 13)	CBS (*N* = 10)	Healthy seniors (*N* = 12)
Age (years)	66 (2)	70 (2)	62 (3)
Education (years)	17 (4)	14 (2)	15 (2)
MMSE score (max = 30)**	25.7 (4.7)	27.2 (2.4)	29.4 (0.7)
Disease duration (years)	2.0 (0.6)	1.0 (0.2)	–
Boston Naming Test (BNT)	26 (1)	27 (3)	–

Notes: **Significant difference between groups: *p* < 0.01; Standard error in parenthesis. Patients and healthy seniors did not significantly differ in age (*F*_(34)_ = 2.696; *p* > 0.08), gender (*X*^2^ = 2.667; *p* > 0.1) and education level (*F*_(34)_ = 2.761; *p* > 0.07). A *post hoc* analysis (Bonferroni correction) revealed that the relatively low p-value in the analysis of age is driven by the contrast between healthy seniors and CBS patients (*p* = 0.081) while in the case of education the difference between bvFTD and CBS patients approach significance (*p* = 0.076). Patients groups differed from healthy seniors in their MMSE score (*F*_(34)_ = 7.564, *p* < 0.01), but a *post hoc* analysis (Bonferroni correction) showed that bvFTD and CBS did not significantly differ in their MMSE score (*p* > 0.1). CBS patient did not significantly differ from bvFTD patients in their BNT total score (*t*_(20)_ = 0.793, *p* > 3) and in the duration of their disease at the time of the test (*t*_(21)_ = 1.360, *p* > 0.1).

### Materials

Eighty nouns were selected, 40 gender-neutral nouns (e.g., “visitor”) and 40 gender-biased nouns (e.g., “boy” or “girl”). The gender of each noun was assessed by a norming procedure described elsewhere (McMillan et al., 2012a). The 80 nouns served as subjects or objects of grammatically simple, active declarative sentences like “The grandmother hugged the groom”. Each noun was repeated no more than six times over the course of the experiment and each noun-noun pair was never repeated. Half of the verbs were alternating-dative (e.g., called) and half reciprocal (e.g., kissed) verbs. Each of these sentences was followed by a two-word pronoun-verb sentence. A total of 120 sentences were generated in order to create 40 stimuli for each of the following conditions:

- *Directly determined* (e.g., “The woman paid the boy. He pouted”): contained 1 female-biased noun and 1 male-biased noun, and the pronoun refers directly only to one of the two possible referents.
- *Indirectly determined* (e.g., “The mom served the child. He pouted”): contained 1 gender-neutral noun and 1 gender-biased noun. The gender of the pronoun does not match the gender of the gender-biased noun, thus the pronoun does not refer to the gender-biased noun and the pronoun indirectly refers to the gender-neutral noun.
- *Ambiguous* (e.g., “The visitor fed the grandfather. He grinned”): there is one gender-biased noun and one gender-neutral noun; the pronoun agrees with the gender-biased noun. In this case there is no strictly correct answer because the pronoun can refers to both names. However, we expected that healthy participant would have found more likely that the referent of the pronoun is the gender-biased noun.

All of the sentences that included a male- or female-biased noun were counterbalanced for gender location (half male as subject, half female as subject). For example, in the Directly Determined condition we presented an equal number of female–male stimulus items (e.g., “woman”–“boy”) and male–female stimulus items (e.g., “boy”–“woman”). The second sentence always contained a past tense verb and the pronoun, and the pronoun was counterbalanced for gender across stimuli. Further details about the generation of the stimuli can be found in McMillan et al. (2012a).

### Behavioral Procedure

All stimuli were displayed visually using a Dell Inspiron 1100 laptop. E-Prime v2.0 presentation software controlled stimulus presentation and recorded response accuracy. Within each trial, the first sentence containing the two nouns (e.g., “The woman paid the boy”), was presented for 3000 ms. Subsequently, the sentence containing the pronoun (e.g., “He pouted”) was added to the visual display beneath the first sentence. We asked participants to choose whether the pronoun refers to the first noun (e.g., “woman”) or the second noun (e.g., “boy”) via button press (with the first noun indicated by a left button press and second noun by a right button press). An equal number of responses for each type of stimulus elicited a left or right button press. No feedback was provided to the participants and they were instructed to guess which of the two noun was the referent of the pronoun. Therefore, participants had to rely only on their own perception of ambiguity and probability. To minimize task-related working memory demands, the linguistic materials presented in each stimulus event remained on the display screen for the full duration of the experimental trial (i.e., until the subject made a choice). Thus, until the completion of their decision, participants had visual access to both the transitive sentence that contained the two nouns and the sentence containing the pronoun.

Prior to the beginning of the main session, participants were presented with 10 practice trials to familiarize them with the structure of the experimental material and with the task. Over the course of the practice trials participants were also allowed to ask clarification questions. These stimulus items were not re-presented in the experimental task. All the 120 stimuli were pseudo-randomized and divided in 5 runs of 30 stimuli each. An equal number of stimuli from each experimental condition were included in each run.

### Volumetric Neuroimaging Procedure and Analysis

High-resolution volumetric T1-weighted MRIs were obtained within an average of 2.4 (±1.2) months from behavioral testing for nine bvFTD patients and seven CBS patients. Reasons for exclusion in the MRI study include issues related to health and safety (e.g., metallic implants, shrapnel, claustrophobia) or lack of interest in participating in an imaging study. High resolution T1-weighted MRI were also collected in a group of 19 healthy seniors who were demographically comparable to the patients group for education (Kruskal-Wallis test = 1.529; *p* > 0.4) and age (Kruskal-Wallis test = 4.545; *p* > 0.1). The healthy seniors who were included in the imaging analysis were not the same who took part in the behavioral study. The computation of the atrophy mask requires a larger number of controls than the behavioral analysis in order to approximate, as close as possible, the neuroanatomy of a typical aging brain.

Images were collected on a Siemens 3.0T Trio scanner with an 8-channel head coil. MRI volumes were acquired using an MPRAGE sequence and the following acquisition parameters: repetition time = 1620 ms; echo time = 3.87 ms; slice thickness = 1.0 mm; flip angle = 15°; matrix = 192 × 256, and in-plane resolution = 1.0 × 1.0 mm. Whole-brain MRI volumes were preprocessed using PipeDream^1^ and Advanced Normalization Tools^2^ using a state-of-the-art procedure described elsewhere (Avants et al., 2008; Klein et al., 2010). Briefly, PipeDream deforms each individual dataset into a standard local template space. A diffeomorphic deformation was used for registration that is symmetric to minimize bias toward the reference space for computing the mappings, and topology-preserving to capture the large deformation necessary to aggregate images into a common space. Template-based priors were used to guide GM segmentation and compute GM probability, which reflects a quantitative measure of GM density. Resulting images were warped into Montreal Neurological Institute (MNI) space, smoothed using a 4 mm full-width half-maximum Gaussian kernel and down-sampled to 2 mm resolution.

Permutation-based imaging analyses were performed with threshold-free cluster enhancement (TFCE; Smith and Nichols, 2009) using the randomize tool in FSL^3^. GM density was compared in patients relative to healthy seniors. Analyses were run with 10,000 permutations and restricted to voxels containing GM using an explicit mask generated from the average GM probability map of all groups. We report clusters that survived a threshold of *p* < 0.01 with family-wise error correction (FWE) for multiple comparisons and contained a minimum of 100 adjacent voxels.

To relate behavioral performance to regions of significant GM disease, we used the randomize tool of FSL with TFCE as described above. An explicit mask restricted the analyses to voxels of GM atrophy in the patients as defined in the group comparison. Permutations were run exhaustively up to a maximum of 10,000 for each analysis. We report clusters surviving a height threshold of *p* < 0.05 TFCE (uncorrected) and a minimum of 30 adjacent voxels. The regression analysis has been applied only to the group of bvFTD patients because their performance differs from the performance of healthy seniors while this was not the case for CBS patients (see the “Results” Section). The main purpose of the imaging analysis was to relate the behavioral difficulties to the underlying brain network that is affected by bvFTD. We leveraged the variance in the individuals’ performance to build a regression model that correlated GMP and the accuracy in the task. However, the investigation of the neural bases of anaphora resolution in participant with a typical behavioral profile (e.g., healthy seniors and CBS patients) would have been beyond the purposes of the present study.

## Results

### Behavioral Results

**Figure 1:**
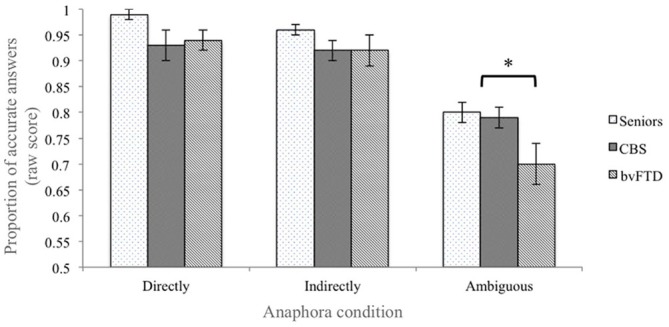
**Performance accuracy in the behavioral task.** Behavioral performance of healthy seniors, corticobasal syndrome (CBS) patients and bvFTD patients. The error bars represent the standard error of the mean. The “*” represents the contrast who reach the statistical significance (see “Results” Section).

Both patient groups and healthy seniors performed near ceiling in the *Directly determined* condition (accuracy (*probability* ± *standard error*): bvFTD patients = 0.94 ± 0.02; CBS patients = 0.94 ± 0.03; healthy seniors = 0.99 ± 0.01). Due to the ceiling effect in this condition, the data were not normally distributed and we conducted all analyses using non-parametric statistics. A Kruskal-Wallis test confirmed that there is no statistical difference among groups in the *Directly determined* condition (Kruskal-Wallis Test = 4.685; *p* > 0.09; see Figure 1). However, considering that *p* > 0.09 is close to significance and that we were analyzing a relatively small sample, an *adjusted accuracy* score was calculated for both the *Indirectly determined* and the *Ambiguous* conditions in order to assure that the performance in the more complex conditions did not reflect any differences in the ability to resolve anaphora reference in the control condition.

Considering, for example, the *Ambiguous* condition, the *adjusted accuracy* was computed as follow: the accuracy in the *Ambiguous* condition was transformed into a *Z* score. The same transformation was applied to the probability of making a mistake (i.e., 1–accuracy) in the *Directed determined* condition. After that, the *adjusted accuracy* was derived by computing the differential score between the *Z* transformed accuracy in the *Ambiguous* condition and the *Z* score of the errors in the *Directed determined* condition. Therefore, the *adjusted accuracy* allowed us to weight the performance in the more complex conditions (e.g., the *Ambiguous* conditions) with the rate of errors at baseline (i.e., the *Directed determined* condition).

In the *Indirectly determined* condition the raw accuracy was still high in all three groups of participants (bvFTD patients = 0.92 ± 0.03; CBS patients = 0.92 ± 0.02; healthy seniors = 0.96 ± 0.01 *(probability ± standard error*); see Figure 1), and the comparison of the *adjusted accuracy* scores showed that the groups did not significantly differ from one another (Kruskal-Wallis Test = 3.190; *p* > 0.2). Together, these findings suggest that bvFTD and CBS are not impaired in the decision task when the linguistic stimulus clearly provides all the necessary information.

In contrast, the analysis of the *Ambiguous* condition revealed a significant difference among groups (Kruskal-Wallis Test = 10.364; *p* < 0.01). In this condition, healthy seniors tended to associate the pronoun with the gender-biased noun (raw accuracy = 0.80 ± 0.02; e.g., in “The visitor fed the grandfather. He grinned”, healthy seniors chose “grandfather” as the referent of “he”). CBS patients followed a pattern similar to healthy seniors and preferred the gender-biased noun over the gender-neutral noun (raw accuracy = 0.79 ± 0.04). bvFTD patients, however, chose the gender-biased noun relatively less often (raw accuracy = 0.70 ± 0.02). Further comparisons between groups revealed that only the performance of the bvFTD patients significantly differed from the performance of healthy seniors (Healthy seniors vs. bvFTD patients: Mann-Whitney *U* = 18.0, *p* < 0.01; Healthy seniors vs. CBS: Mann-Whitey *U* = 34.0, *p* > 0.09; bvFTD vs. CBS: Mann-Whitney *U* = 47.5, *p* > 0.2; see Figure 1).

### Imaging Results

**Figure 2:**
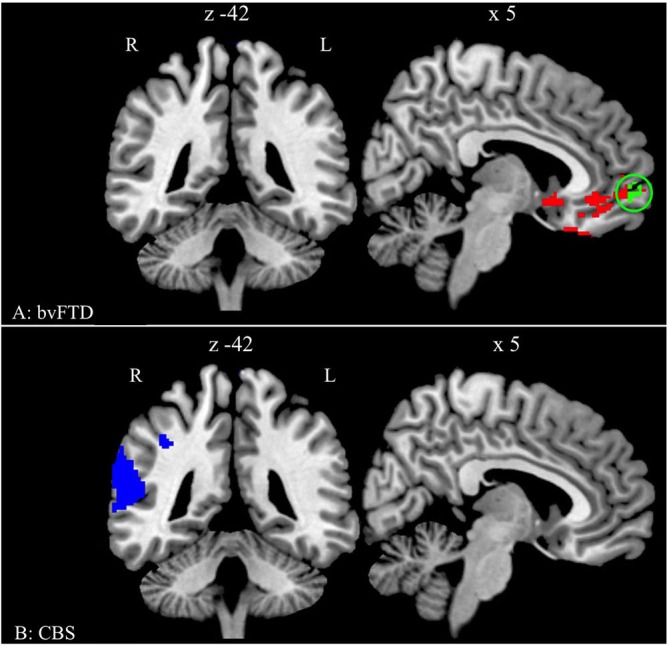
**Significant gray matter (GM) atrophy in behavioral variant frontotemporal degeneration (bvFTD) and CBS, and regression analysis relating performance to reduced GM density. (A)** Regions of reduced GM density in patients with bvFTD relative to healthy seniors (red and green) and regions related to the *adjusted accuracy* of ambiguous anaphoric reference in bvFTD patients (green). The green cluster is in the ventromedial prefrontal cortex (vmPFC) (BA 10/11; peak: 2 60 −8). **(B)** Regions of reduced GM density in patients with CBS relative to healthy seniors (blue).

The analysis of GM density in bvFTD patients relative to healthy seniors revealed significant atrophy in medial and orbital frontal regions and in the right insula (threshold of *p* < 0.01 with FWE for multiple comparisons and contained a minimum of 100 adjacent voxels; see Table 2 and Figure 2A). The comparison between healthy seniors and CBS patients revealed cortical atrophy in the right superior and inferior parietal lobe and extending into the superior temporal lobe (threshold of *p* < 0.01 with FWE for multiple comparisons and containing a minimum of 100 adjacent voxels; see Table 2 and Figure 2B). We related the *adjusted accuracy* score in the *Ambiguous* condition to GM atrophy in the bvFTD cohort using a regression analysis. The results revealed a significant association of the *adjusted accuracy* with GM density in vmPFC (Brodmann area (BA) 10) (height threshold of *p* < 0.05 TFCE (uncorrected) and a minimum of 30 adjacent voxels; see Table 2 and Figure 2A).

**Table 2:** **(A) Regions with reduced gray matter (GM) density in the bvFTD cohort respect to the group of healthy seniors; (B) Regions with reduced GM density in the CBS cohort respect to the group of healthy seniors; (C) Regions of reduced GM density in patients with bvFTD that relate to the adjusted accuracy**.

				MNI coordinates		
	L/R	*K*	*p* value	*x*	*y*	*z*
**A: Reduced GM (BA) bvFTD < Healthy seniors**
Medial prefrontal cortex and medial orbitofrontal cortex (10–11)	L/R	832	0.002	20	14	−2
Inferior frontal gyrus pars orbitallis (47)	R	102	0.008	30	16	−27
**B: Reduced GM (BA) CBS < Healthy seniors**
Inferior parietal cortex (40)	R	3017	0.001	43	−48	49
Superior/middle temporal gyrus (48–22)			0.001	62	−56	18
**C: Results of the regression analysis in the bvFTD cohort (BA) based on an explicit mask of 934 voxels**
Ventromedial prefrontal cortex (10/11)	L/R	34	0.001	2	60	−8

*Note: BA, Brodmann area; MNI, Montreal Neurological Institute*.

## Discussion

In the present report, we investigated the cognitive and neuroanatomic basis for solving a simple decision-based task requiring the ability to identify the most likely referent of a pronoun in ambiguous contexts. bvFTD patients exhibited a clear limitation in the *Ambiguous* condition. Because bvFTD patients do not have obvious aphasic symptoms thus, any difference in performance between healthy seniors and bvFTD patients cannot be easily attributed to language-related deficits. A group of CBS patients, who show atrophy of parietal cortex, was also tested with the same materials in order to investigate the extent to which bvFTD patients’ limitations can be specifically related to the degeneration of the prefrontal cortex that characterizes this condition.

The results showed that both patient groups are able to correctly identify the antecedent of a pronoun when an unambiguous referent is available purely on the basis of the linguistic code. In both the *Directly determined* and the *Indirectly determined* conditions, the gender of the pronoun is compatible with only one of the two available antecedents, and therefore the linguistic stimulus provided enough information to resolve the anaphoric reference.

It is important to point out that the difference between the patient groups and the healthy seniors approaches significance in the *Directly determined* condition. However, both bvFTD and CBS patients perform well in this condition, with a mean accuracy of 94%. Therefore, it seems likely the relatively low *p*-value (*p* > 0.09) is due to almost perfect performance found in healthy seniors (mean accuracy = 99%). Regardless, the *adjusted accuracy* was computed for both the *Indirectly determined* and the *Ambiguous* conditions in order to take the errors in the *Directly determined* condition into account.

In the *Ambiguous* condition, both nouns presented in the first sentence could theoretically be the referent of the pronoun presented in the second sentence. In this case, the decision process is more demanding because it requires participants to extrapolate information that is not explicitly stated in the linguistic stimulus itself. Instead, the participants must evaluate the differential likelihood that the pronoun refers to one of the two alternatives, arguably on the basis of previous experiences (e.g., background knowledge about the use of anaphoric pronouns in daily conversations). For healthy seniors, the gender-biased noun appeared to be a more plausible referent than the gender-neutral noun, arguably because we tend to assume that speakers are cooperative and try to communicate effectively (see e.g., Grice, 1975; Sperber and Wilson, 1986; Clark, 2011; for a similar argument).

Cortical thinning in the vmPFC may prevent bvFTD patients from including the evaluations of the differential likelihood that the pronoun refers to one of the two alternatives. bvFTD patients chose, indeed, the gender-biased noun significantly less often than healthy seniors. This hypothesis is to some extent speculative and further work is needed to test which aspect of decision-making is mostly affected by bvFTD causing the difficulties in anaphora resolution and in processing ambiguous stimuli more in general.

Regardless of the specific basis for difficulty with ambiguous references, our data do not appear to support findings implicating the parietal lobe in resolving linguistic ambiguities. BOLD fMRI studies have suggested that parietal regions are recruited during anaphora resolution (Almor et al., 2007; Nieuwland et al., 2007; McMillan et al., 2012a). In the present study, the performance of CBS patients, who have reduced GM density in the parietal cortex, did not differ from the performance of healthy seniors. One possible explanation for this apparent puzzle is that fMRI studies have predominantly shown activations in bilateral parietal regions while the cohort of CBS patients tested here showed significant GM atrophy only in the right parietal lobe. Thus it is possible that left parietal region contributes to processing ambiguous anaphoric reference. Furthermore, the present study is based on an off-line measure and thus is not well suited for detecting subtle difference between groups that might be captured by on-line measures. However, BOLD studies highlight regions that may be involved in performing a task but do not identify regions that are truly *necessary* for a given process. Additional work is needed to specify the precise role of the parietal lobe in anaphora and ambiguity resolution. Future studies will also look to the correlation between GMP and anaphora resolution across the border of clinical categorization to investigate on a broader scale the relationship between cortical thickness and the processing of ambiguity in the linguistic domain.

Several other shortcomings should be kept in mind when considering this study. First, due to the difficulty of recruiting patients who meet strict clinical criteria, we examined relatively small samples of bvFTD and CBS patients. Next, although we used a simple, untimed procedure, bvFTD patients have executive deficits that may interfere with task performance. While bvFTD patients do not have obvious difficulties with phonological, semantic, or syntactic aspects of language, some work has shown deficits on language measures such as narrative comprehension (Farag et al., 2010) and grammatical comprehension (Charles et al., 2014).

## Conclusion

With these caveats in mind, the results of the present study seem to show that bvFTD patients have difficulties in decision processes that are involved in the resolution of linguistic ambiguities. Although sentence pairs like (2) “John kissed the visitor. He smiled” can be consider extreme cases in real communication, especially if they are not embedded in a larger context, anaphoric pronouns as well as homonyms and linguistic structures involving some degree of ambiguity are fairly common in daily conversations. Future works employing direct measures of language processing and exploring other case of ambiguity will help to clarify to what extent bvFTD patients’ limitation in decision making can interfere with the understanding of ambiguity in language.

## Conflict of Interest Statement

The authors declare that the research was conducted in the absence of any commercial or financial relationships that could be construed as a potential conflict of interest.

## Footnotes

^1^https://sourceforge.net/projects/neuropipedream/

^2^http://www.picsl.upenn.edu/ANTS/

^3^http://fsl.fmrib.ox.ac.uk/fsl/fslwiki
